# Unmasking Heavily *O*-Glycosylated Serum Proteins Using Perchloric Acid: Identification of Serum Proteoglycan 4 and Protease C1 Inhibitor as Molecular Indicators for Screening of Breast Cancer

**DOI:** 10.1371/journal.pone.0149551

**Published:** 2016-02-18

**Authors:** Cheng-Siang Lee, Nur Aishah Mohd Taib, Ali Ashrafzadeh, Farhana Fadzli, Faizah Harun, Kartini Rahmat, See Mee Hoong, Puteri Shafinaz Abdul-Rahman, Onn Haji Hashim

**Affiliations:** 1 Department of Molecular Medicine, Faculty of Medicine, University of Malaya, Kuala Lumpur, Malaysia; 2 Department of Surgery, Faculty of Medicine, University of Malaya, Kuala Lumpur, Malaysia; 3 Medical Biotechnology Laboratory, Faculty of Medicine, University of Malaya, Kuala Lumpur, Malaysia; 4 Department of Biomedical Imaging, Faculty of Medicine, University of Malaya, Kuala Lumpur, Malaysia; 5 University of Malaya Centre for Proteomics Research, Faculty of Medicine, University of Malaya, Kuala Lumpur, Malaysia; Universidade de São Paulo, BRAZIL

## Abstract

Heavily glycosylated mucin glycopeptides such as CA 27.29 and CA 15–3 are currently being used as biomarkers for detection and monitoring of breast cancer. However, they are not well detected at the early stages of the cancer. In the present study, perchloric acid (PCA) was used to enhance detection of mucin-type *O*-glycosylated proteins in the serum in an attempt to identify new biomarkers for early stage breast cancer. Sensitivity and specificity of an earlier developed sandwich enzyme-linked lectin assay were significantly improved with the use of serum PCA isolates. When a pilot case-control study was performed using the serum PCA isolates of normal participants (n = 105) and patients with stage 0 (n = 31) and stage I (n = 48) breast cancer, higher levels of total *O*-glycosylated proteins in sera of both groups of early stage breast cancer patients compared to the normal control women were demonstrated. Further analysis by gel-based proteomics detected significant inverse altered abundance of proteoglycan 4 and plasma protease C1 inhibitor in both the early stages of breast cancer patients compared to the controls. Our data suggests that the ratio of serum proteoglycan 4 to protease C1 inhibitor may be used for screening of early breast cancer although this requires further validation in clinically representative populations.

## Introduction

Mucins are heavily glycosylated proteins containing predominantly *O-*linked oligosaccharide chains that are covalently attached to serine or theorine residues of their polypeptide backbones. They are often present at many epithelial surfaces of the body, including mammary glands as well as gastrointestinal, genitourinary and respiratory tracts [1]. The unique feature of these mucin-type O-glycosylated proteins is that glycosylation begins with addition of N-acetylgalactosamine (GalNAc) by a large family of UDP-GalNAc:polypeptide N-acetylgalactosaminyltransferases [2]. Further elongation with attachment of additional monosaccharides onto O-GalNAc residue is catalysed by 30 or more different glycosyltransferases [3] and the process is usually terminated by addition of blood type antigen, fucose, or sialic acid. Incomplete mucin O-glycosylation is known to generate truncated glycan structures which are present on the surface of cancer cells [4, 5]. These heavily glycosylated proteins often get actively secreted or shed, causing their increased levels in the blood circulation. High levels of mucins in patients with breast cancer correlate with poor prognosis and increasing tumour burden [6].

The cancer antigens CA 27.29 and CA 15–3, which are derived from a mucin known as MUC-1, are commonly used biomarkers for breast cancer [7, 8]. Whilst CA 27.29 is a soluble form of the MUC-1 glycoprotein, CA 15–3 is a transmembrane form of the glycoprotein that is released from breast cancer cells into the blood circulation. Because of its strong association with breast cancer progression, CA 27.29 is currently used as a biomarker for monitoring of breast cancer. On the other hand, CA 15–3 which is detected in more advanced breast cancer patients [9], is used in evaluation of recurrence of the disease. Although the use of these mucin-type glycoproteins in clinical practice has generally improved monitoring of breast cancer, their detection is rather poor at the early stages of the cancer [3], and hence not applicable as a breast cancer screening biomarker.

We have previously reported the development of a sandwich enzyme-linked lectin assay (ELLA) to evaluate trace amounts of mucin-type *O-*glycoproteins from serum samples [10]. The assay utilizes pre-coated champedak galactose binding (CGB) lectin to capture *O-*glycosylated proteins from serum samples, followed by binding of biotinylated-jacalin as a lectin probe. The aim of this study was to use perchloric acid to improve detection of heavily *O*-glycosylated serum proteins using lectin-based method for the purpose of identifying potential tumour markers. Since perchloric acid (PCA) is known to solubilize heavily glycosylated proteins from serum [11, 12], we have used it to further enrich the glycoproteins in serum samples prior to application of ELLA in the present study. In addition, we have also analysed the total levels of *O-*glycosylated proteins in serum samples of patients with stage 0 and stage I breast cancer as well as those from controls or cancer free women using the method and determined their glycoprotein compositions using gel-based proteomics. By comparing the PCA-enriched glycoprotein profiles of patients with pre-invasive and invasive breast cancer with those of the controls, we were able to identify glycoproteins of altered abundance that may be used as potential biomarkers for detection of the early breast cancer.

## Materials and Methods

### Sample collection

A case control study was done matching cases to controls by age and ethnicity. Cases were recruited consecutively between April 2013 to December 2014, clinical data and bio-specimens were collected prospectively as part of the University Malaya Medical Centre (UMMC) breast cancer clinical and biospecimen repository. A total of 79 consecutive pre-treatment serum samples of stage 0 and I breast cancer patients were recruited into the study. Among these, 31 were pathologically confirmed as stage 0 (carcinoma in situ) while 48 were diagnosed with stage I breast cancer. The controls were recruited from well women attending opportunistic breast mammogram at the Department of Biomedical Imaging and their mammogram showed no indications of abnormalities (BIRADS 1) from April 2014 to Dec 2014. The rationale for selecting these groups of patients was to study the differences in the protein profiles of serum PCA isolates of pre-invasive, invasive and normal subjects. Differences seen within the groups of early breast cancer patients and cancer free subjects could possibly lead to development of a method to screen for breast cancer. Written informed consent was obtained from all participated subjects prior to sample collection. This study and its consent procedure were approved by the Medical Ethics Committee of the University of Malaya Medical Centre (MEC No. 1004.17).

### Enrichment of serum glycoproteins using PCA

Enrichment of glycoproteins was performed by treating the serum samples with perchloric acid (PCA) at a final concentration of 0.6 N. This concentration was chosen based on the results of our optimization study conducted using four different normalities of PCA. This same PCA normality has also been used in earlier studies of serum mucoproteins [11]. Fifty μl of serum, unless otherwise stated, was incubated with an equal volume of cold PCA for 20 min. Supernatant was collected after centrifugation at 10,000 x g for 10 min and neutralized by adding sufficient amount of 1.0 N KOH, with phenol red as the pH indicator. Perchlorate salt was removed by centrifugation at 10,000 x g for 10 min. The supernatant was collected and protein concentration was estimated using the BCA protein microassay (Thermofisher Scientific, Rockford, IL, USA). Serum PCA isolates obtained were finally subjected to dialysis and lyophilisation prior to their subsequent analyses.

### Trypsin digestion and mass spectrometry

Lyophilised protein samples were reconstituted in 37 μl of 50 mM ammonium bicarbonate with 4 mM of DTT. The samples were reduced at 95°C for 5 min. Alkylation was performed by incubating 7.5 mM iodoacetamide at room temperature in dark for 20 min. The protein sample was digested using 0.3 μg of Trypsin Gold (Promega, Madison, WI, USA) for 16 hours at 37°C. The reaction was quenched by freezing the sample. Digested peptides were desalted using μ-C_18_ ZipTips (Billerica, MA, USA) and eluted in 50% acetonitrile containing 1% formic acid. The peptides were subjected to QTOF LC/MS analysis using Agilent 6550 iFunnel QTOF LC/MS system (Agilent, Santa Clara, CA, USA) for peptides identification. Tryptic peptides were separated using the C18 HPLC-Chip with a linear gradient of water/acetonitrile. Spectra were analysed to identify proteins of interest according to the Uniprot database.

### Biotinylation of asialo-BSM

Bovine submaxillary mucin (BSM) was prepared from fresh glands as previously reported by Variyam et al. [13]. BSM was chemically desialylated by mild acid hydrolysis (asialoBSM). Biotinylation was performed according to the manufacturer’s instruction. Briefly, a sufficient volume of 10 mg/ml EZ-Link sulfo-NHS-biotin reagent (Thermofisher Scientific, Rockford, IL, USA) was added to a solution of asialoBSM (5 mg/ml in PBS). The solution was mixed gently at room temperature for 60 min. The reaction mixture was dialyzed for 48 hours with two changes of distilled water. The amount of biotinylated-asialoBSM recovered in the retentate was determined using BCA protein microassay (Thermofisher Scientific, Rockford, IL, USA).

### Biotinylated-asialo-BSM spiking experiment

Five μg of biotinylated-asialoBSM was spiked into serum samples, followed by PCA treatment as earlier described. A theoretical control was prepared by addition of 5 μg of biotinylated-asialoBSM into serum after PCA treatment. Levels of biotinylated-asialoBSM were measured using the following protocol: Microtiter plate (Jet-Biofill, Guangzhou, China) was coated overnight with 50 μl of 1 μg champedak galactose binding (CGB) lectin per well. The CGB lectin, which specifically binds to *O*-linked oligosaccharide chains, was extracted and purified from the seeds of champedak in the laboratory as previously described [14, 15]. The plate was washed thrice with washing buffer containing 0.01 M Tris-HCl, pH 8.0, 0.05% Tween-20 and 0.05% sodium azide. Biotinylated-asialoBSM spiked samples were added to the precoated wells at dilutions ranging from 1:5,000 to 1:160,000. Biotinylated-asialoBSM, at concentrations ranging from 10 to 0.156 ng/ml, was used as standard.

### Sandwich enzyme-linked lectin assay

Sandwich enzyme-linked lectin assay (ELLA) was performed as previously described [10]. All serum samples and serum PCA isolates were subjected to chemical desialylation by mild acid hydrolysis (0.1 N sulfuric acid, 80°C, 60 min) prior to estimation of *O*-glycosylated proteins by ELLA. Briefly, a 96-well microtiter plate was pre-coated with purified CGB lectin in coating buffer (0.1 M sodium carbonate, pH 9.6) at 4°C overnight. The wells were washed thrice with TBS-T (0.01 M Tris-HCl, pH 8.0, 0.05% Tween-20 and 0.05% sodium azide) and blocked with the buffer for 30 min. Fifty μl of sample in PBS was added to each well and incubated for 90 min at room temperature. Unbound materials were removed by washing as earlier described. Fifty μl of biotinylated-jacalin (1 μg/ml; E-Y Labs, San Mateo, CA, USA) was added to each well and incubated for 90 min at room temperature. Plate was again similarly washed. Fifty μl of avidin alkaline phosphatase (1:10000) in TBS-T was added to the wells and incubated for 90 min. The plate was washed thrice before adding 50 μl of 1 mg/ml p-nitrophenyl phosphate in substrate buffer (0.1 M sodium carbonate, pH 9.6, 1 mM MgCl_2_). Absorbance was read at 415 nm using a microplate reader (Bio-Rad, USA) after an appropriate incubation time of typically 20–30 min at 20°C. AsialoBSM was used as the *O*-glycosylated protein standard. The coefficients of variation of intra- and inter-assay were estimated as 5.90% and 9.06%, respectively.

### Conjugation of CGB lectin

Purified CGB lectin was conjugated to CNBr activated Sepharose 4B according to the manufacturer’s protocol (Sigma Chemical, St. Louis, MO, USA). About 5 μl of Sepharose conjugated CGB lectin was transferred into a 1.5 ml microcentrifuge tube. The beads were washed three times by adding 1 ml of wash buffer (0.01 M Tris-HCl, pH 8.0, 0.05% Tween-20 and 0.05% sodium azide) with continuous vortex for 10 min. The wash buffer was removed by brief centrifugation. The beads were blocked with wash buffer for at least 30 min with continuous vortex.

### Isolation and Profiling of *O*-glycosylated Proteins

Two hundred and fifty μl of serum PCA isolate (120 μg/ml) was added to the Sepharose conjugated CGB lectin beads and incubated for 90 min at room temperature with constant vortex. Unbound proteins were removed by centrifugation and the beads were washed as earlier described. The CGB lectin bound proteins were released by adding 25 μl of Laemmli buffer and incubated at 95°C for 5 min. Released peptides were resolved in 8% SDS-polyacrylamide gel (Biorad, Hercules, CA, USA). Peptide bands were visualized by silver staining. The developed gel was scanned using ImageScanner III (GE Healthcare, Little Chalfont, BU, UK) and image analysis was performed using GelAnalyzer software. Each of the peptide bands was excised and subjected to typical in-gel trypsin digestion [16]. The extracted peptides were analysed using Agilent 6540 mass spectrometer (Agilent, Santa Clara, CA, USA). Proteins were identified by subjecting the spectra to Mascot sequence matching software (Matrix Science) analysis using the Ludwig NR database.

### Western blot

Proteins from a duplicate SDS-polyacrylamide gel were transferred onto nitrocellulose membrane using a mini trans-blot system (Biorad, CA, USA) for 1 hour at a constant voltage of 100 V. Standard Towbin buffer without SDS (25 mM Tris, 192 mM glycine, 20% methanol) was used as transfer buffer. The membrane was blocked with 5% skim milk in TBST (20 mM Tris, 500 mM NaCl, 0.05% Tween, pH 7.5) for 30 min and washed at least thrice with TBST. Rabbit anti-plasma C1 inhibitor (Abcam, CBG, UK) was added and allowed to bind at 4°C overnight. Horse radish peroxidase conjugated secondary goat anti-rabbit (Abcam, CBG, UK) was added after washing with TBST and incubated for 1 hour. The membrane was washed thrice before being developed using 3,3’-diaminobenzidine (Thermofisher, MA, USA).

### Statistical Analysis

All data was expressed in mean ± SEM, unless otherwise stated. Statistical analysis was performed using IBM SPSS statistical software version 21 (IBM, New York, USA). Student t-test was used for comparison between groups. Receiver operative characteristic (ROC) was performed to evaluate the ELLA performance to detect stage 0 and stage I breast cancer. *P* values of less than 0.05 were considered significant.

## Results

### Identification of proteins in serum PCA isolates

Fourteen proteins were successfully identified when normal serum PCA isolates were analysed by QTOF LC/MS. These isolates were initially subjected to dialysis and lyophilisation, followed by in-solution trypsin digestion. The list of proteins identified by QTOF LC/MS analysis is shown in Table 1. Almost all the detected proteins are known to be glycosylated. Among these, plasma protease C1 inhibitor, hemopexin, protein AMBP, and complement C4-A were identified as proteins that are known to be *O-*glycosylated. Twelve others were previously documented as *N-*glycosylated proteins.

**Table 1:** List of proteins present in serum PCA isolates.

Accession No.	Protein Name	MS/MS Search Score	No. of Matched Sequence	Sequence Coverage (%)	Theoretical Mass (Da)	Glyc-an	Ref.
P05155	Plasma protease C1 inhibitor	194.37	10	26.6	55381.4	*N*- *O*-	[39, 41, 42]
P01009	Alpha-1-antitrypsin	174.65	9	31.1	46906.8	*N*-	[43–45]
P02763	Alpha-1-acid glycoprotein 1	104.45	5	27.8	23739.3	*N*-	[44–46]
P02749	Beta-2-glycoprotein 1	96.15	5	27.5	39609.7	*N*-	[44, 45]
P00734	Prothrombin	81.88	4	10.4	71519.0	*N*-	[43–45]
P02790	Hemopexin	79.74	4	12.7	52417.1	*N*- *O*-	[41, 47]
P02766	Transthyretin	76.93	4	48.2	16000.8	*N*-	[44]
P02760	Protein AMBP	55.81	3	11.3	39911.7	*N*- *O*-	[43]
P06727	Apolipoprotein A-IV	45.18	2	7.3	45398.2	-	-
P19652	Alpha-1-acid glycoprotein 2	40.12	2	14.4	23887.5	*N*-	[44–46]
P0C0L4/ P0C0L5	Complement C4-A or Complement C4-B	34.82	2	0.8	194379.5	*N*- *O*-	[41, 44, 45]
P08185	Corticosteroid-binding globulin	19.52	1	3.7	45311.2	*N*-	[44, 45]
P12259	Coagulation factor V	18.51	1	0.5	252839.9	*N*-	[44]
P02775	Platelet basic protein	17.09	1	7	14179.2	-	-

### Determination of *O-*glycosylated proteins in serum PCA isolates

**Fig 1:**
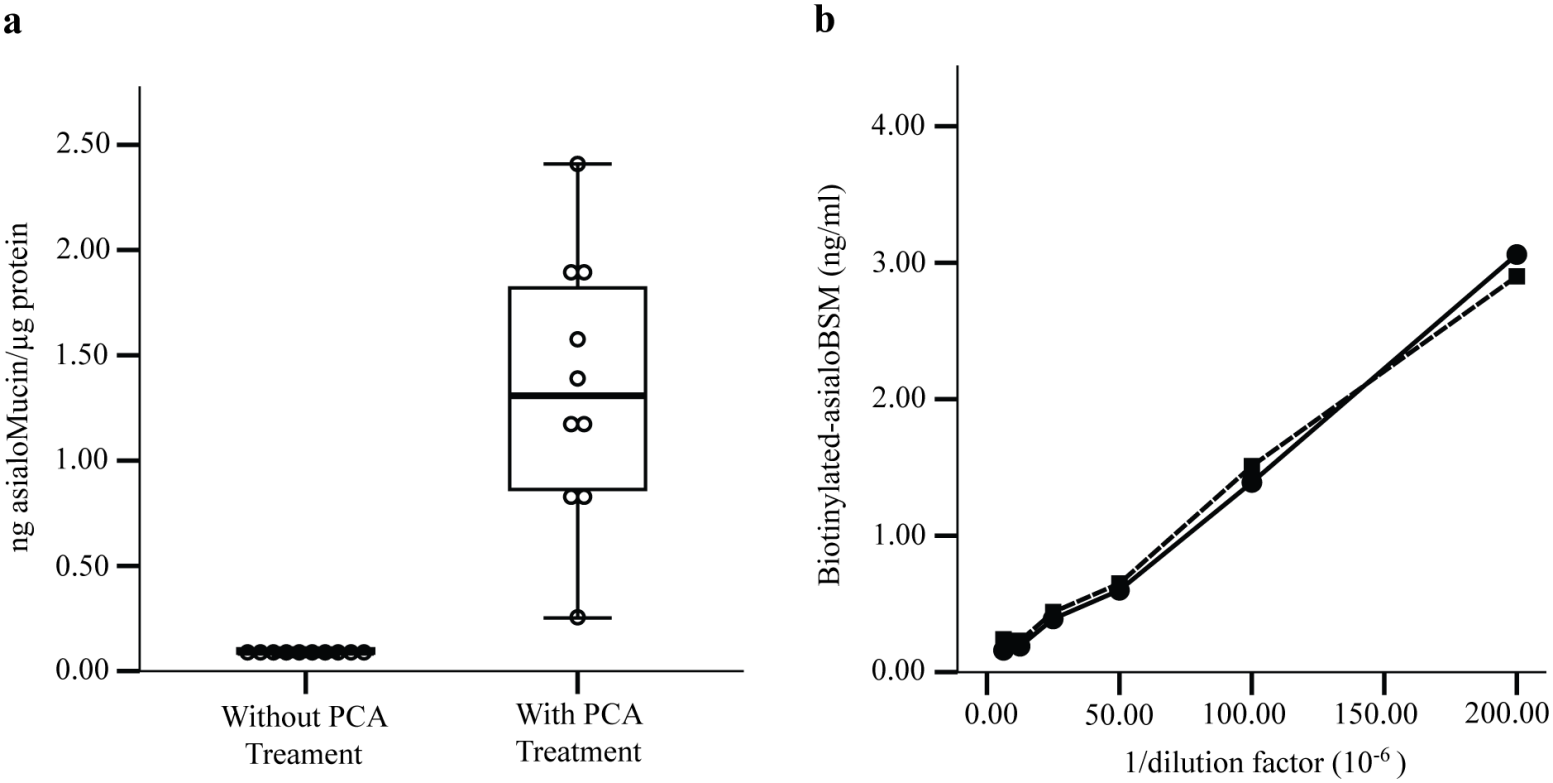
Levels of *O*-glycosylated proteins in serum perchloric acid (PCA) isolates. (a) The levels of *O*-glycosylated proteins were estimated using sandwich ELLA with asialoBSM as the standard *O*-glycosylated protein. (b) *O*-glycosylated proteins recovery assay was performed by spiking biotinylated-asialoBSM into serum samples and followed by PCA treatment. (●) Biotinylated-asialoBSM spiked into serum samples followed by PCA treatment; (■) Biotinylated-asialoBSM spiked into serum PCA isolates.

To analyse the capability of PCA in enriching *O-*glycosylated proteins from human serum, levels of the *O-*glycosylated proteins were determined using a sandwich ELLA. The lectin assay was performed on ten random normal human serum samples that were both treated and not treated with PCA, and the levels of the *O-*glycosylated proteins were expressed in ng asialoMucin/μg protein (Fig 1, panel (a)). Our data suggests that PCA was capable of enriching approximately 14.6-fold amounts of *O-*glycosylated proteins from the serum samples.

In another related experiment, we have assessed whether PCA would have any effect on the recovery of *O-*glycosylated- and biotinylated-asialoBSM that was spiked into serum. In this experiment, 5 μg of biotinylated-asialoBSM was spiked into 50 μl of serum samples before and after PCA treatment. To detect the spiked biotinylated asialo-BSM, we employed a technique similar to the earlier described sandwich ELLA in which serum samples were first allowed to be captured by CGB lectin that was precoated onto plates. This was followed by binding of streptavidin-conjugated alkaline phosphatase. Due to presence of other *O-*glycosylated proteins in the serum PCA isolates, not all asialoBSM was expected to bind to the CGB lectin. Panel (b) of Fig 1 shows the amounts of spiked biotinylated-asialoBSM recovered from serum before and after treatment with PCA, which were not significantly different. Our result also indicated that about 92.1% of biotinylated-asialoBSM was successfully retained. This suggests that PCA was highly effective in enriching the heavily glycosylated proteins from serum.

### Analysis of *O-*glycosylated proteins in sera of patients with stage 0 and stage I breast cancer

**Fig 2:**
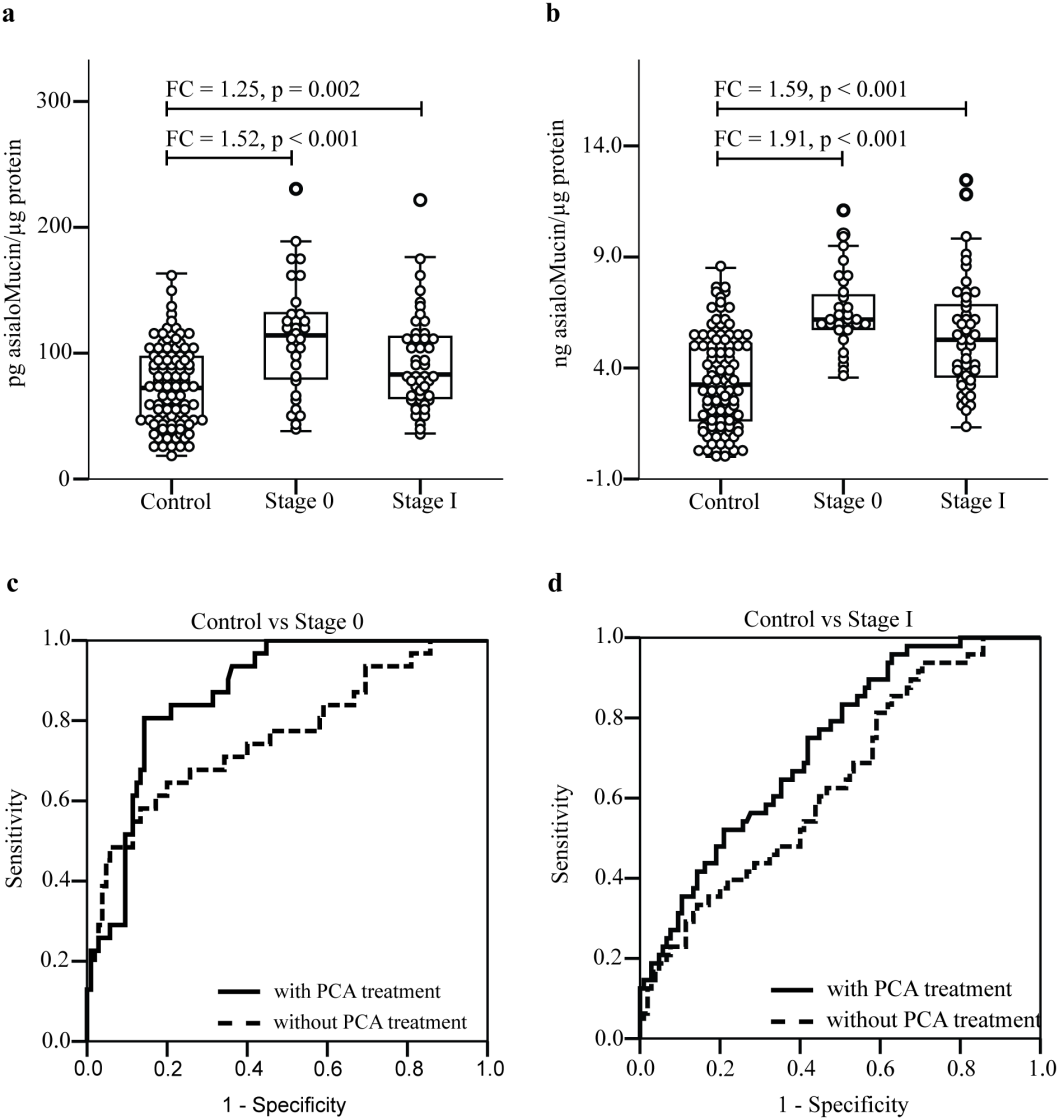
Estimation of total *O*-glycosylated protein in serum samples of cancer-free women (control) and patients with early stages of breast cancer. Serum samples were analysed by sandwich ELLA without (a) and after PCA treatment (b). The assay performance was evaluated by performing Receiver Operative Characteristic analysis on the level of *O*-glycosylated protein detected before and after serum treated with PCA. (c) and (d) refer to analysis of stage 0 and stage I serum samples, respectively.

Using the earlier described sandwich ELLA, we have evaluated the levels of *O-*glycosylated proteins in serum samples of patients with stage 0 (n = 31) and stage I (n = 48) breast cancer as well as those from the controls (n = 105). There was no statistical difference in the distribution of age and ethnic groups in the three groups. Serum samples of breast cancer patients of both stages showed significantly higher levels of *O-*glycosylated proteins compared to those of normal subjects (Fig 2, panel (a)). When the experiment was repeated on the same serum samples but were subjected to PCA treatment, relatively higher levels of *O-*glycosylated proteins were detected in all subjects. Similar differences in the levels of *O-*glycosylated proteins between controls and patients with stage 0 and stage I breast cancer were also observed (Fig 2, panel (b)). A receiver operative characteristic (ROC) analysis was subsequently performed to evaluate performance of the assay in detecting the *O-*glycosylated proteins in serum samples of the breast cancer patients. Assay performed on non-PCA treated stage 0 serum samples had an overall accuracy of 0.760 area under the curve (AUC) whilst increased accuracy of detection of up to 0.869 AUC was observed in assay performed using the same serum samples that were treated with PCA (Fig 2, panel (c)). Similarly, PCA treatment of serum improved the assay detection of stage I breast cancer from 0.638 AUC to 0.730 AUC (Fig 2, panel (d)). In case of stage 0, treatment with sera with PCA improved the sensitivity and specificity of detection from 68% to 81% whilst for stage I breast cancer, the sensitivity and specificity of the assay rose from 56% to 65%.

### Interaction of serum PCA isolates with CGB lectin

**Fig 3:**
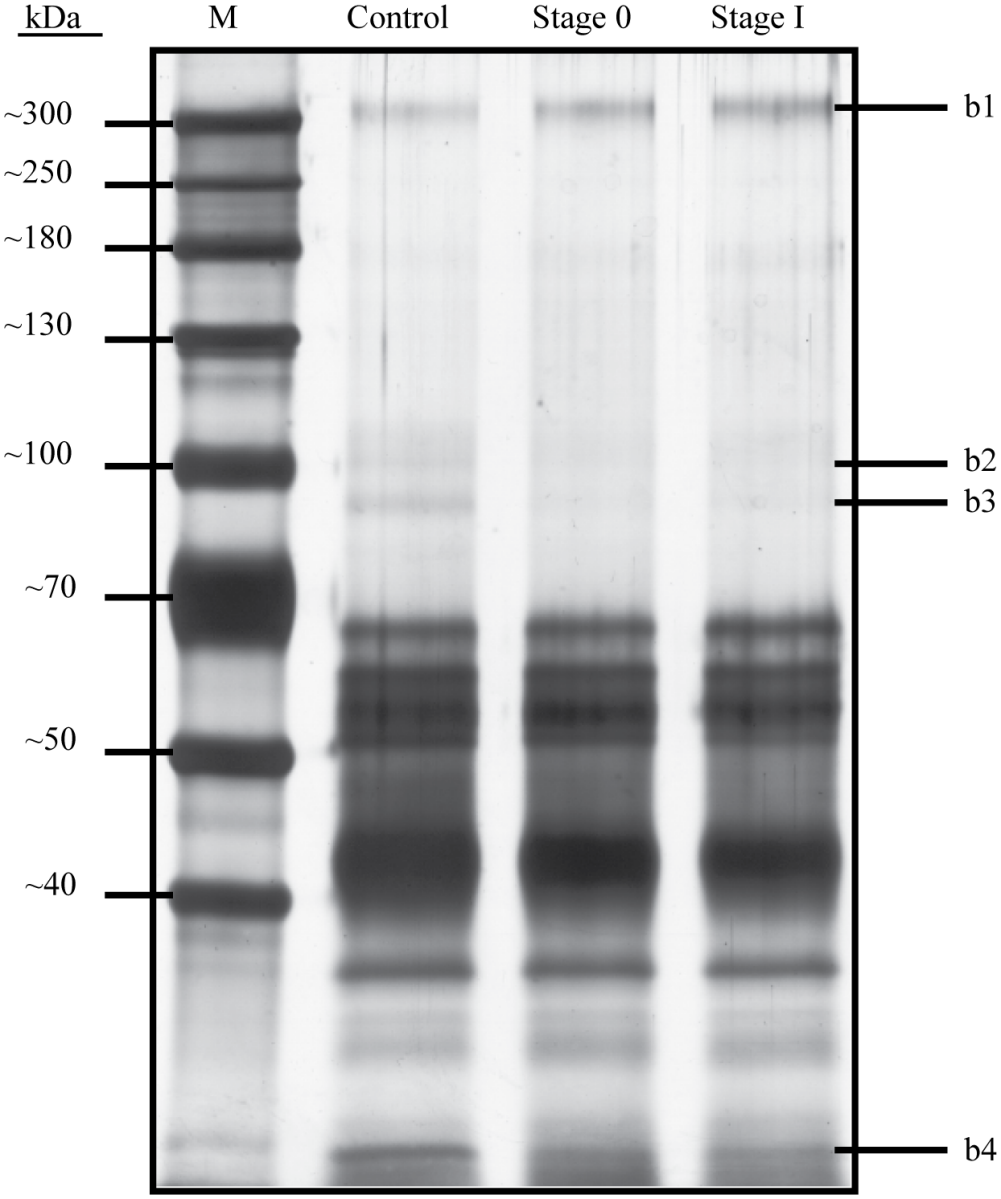
Profiles of CGB lectin bound proteins of normal subjects and breast cancer patients. CGB lectin conjugated to Sepharose was allowed to interact with serum PCA isolates of cancer-free women (control) and patients with stage 0 and stage I breast cancer. Bound proteins were released with Laemmli buffer and resolved in 8% SDS-polyacrylamide gel, which was stained with silver. The gel image was analysed using GelAnalyzer software.

In this study, we have adopted a method to capture *O-*glycosylated proteins from serum samples without the use of column chromatography. This method uses CGB lectin that was conjugated to Sepharose beads. Five μl of the CGB-Sepharose beads were initially allowed to interact with serum PCA isolates and the beads were then washed extensively with washing buffer to remove unbound proteins. Bound *O-* glycosylated proteins were subsequently released using Laemmli buffer and they were resolved by electrophoresis in 8% SDS-polyacrylamide gel. When the CGB lectin precipitation was performed on pooled serum PCA isolates of patients with stage 0 (n = 31) and stage I (n = 48) breast cancer as well as those of controls (n = 105), different protein profiles were generated (Fig 3). Some of the protein bands appeared to demonstrate differences in intensities between pooled sera of both Stage 0 and I breast cancer patients and those that were generated from controls.

### Identification of PCA isolated proteins of altered abundance

**Fig 4:**
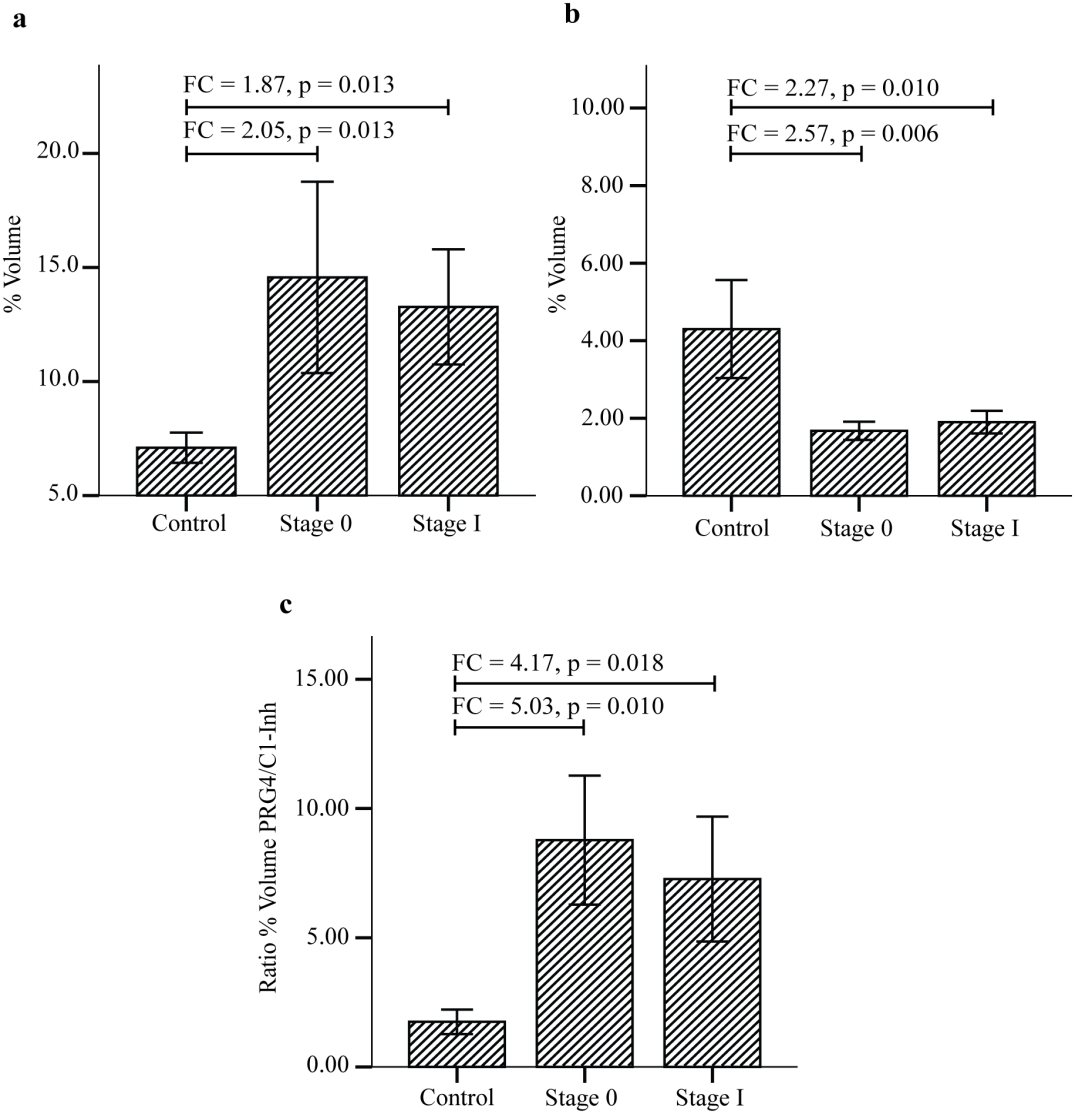
Levels and ratio of CGB lectin bound proteins in normal control women and breast cancer patients. Percentage of volume was obtained using GelAnalyzer software. The percentage volume of each peptide band was compared using SPSS. (a) and (b) refer to mean % volumes of proteoglycan 4 and plasma protease C1 inhibitor, respectively. (c) The ratio of proteoglycan 4/plasma protease C1 inhibitor (PRG4/C1-inh) demonstrates higher fold-change difference between both the early stages of breast cancer compared to controls.

When the SDS-PAGE serum protein profiles of controls were subjected to densitometry and compared with those of patients with both stage 0 and stage I breast cancer, only four protein bands appeared to demonstrate more than 1.5-fold difference of intensities. To identify these proteins of interest, the highly resolved bands were excised in accordance to those marked in Fig 3 and subjected to mass spectrometry analysis. Our database search identified the largest protein (b1) resolved by the SDS-PAGE as that of proteoglycan 4 (also known as lubricin), and two other protein bands that showed differences of abundance were those of plasma protease C1 inhibitor (Table 2). However, the fourth protein band of interest (b4) was not affirmatively identified probably due to presence of multiple proteins. When densitometry was reanalysed based on identities of the proteins, the total levels of plasma protease C1 inhibitor (b2 and b3) of stage 0 and stage I breast cancer patients were both significantly reduced compared to the controls (Fig 4, panel (b)). On the contrary, the abundance of proteoglycan 4 (b1) was significantly higher in both stage 0 and stage I breast cancer patients (Fig 4, panel (a)). Because of the reciprocal difference in levels of proteoglycan 4 and plasma protease C1 inhibitor between both groups of breast cancer patients compared to the controls, calculated ratios of abundances of the two serum proteins further amplified their differences, with more than 5-fold difference for stage 0 and 4.17-fold difference in case of stage I (Fig 4, panel (c)). This is suggestive of the strong potential application of the ratio of the two serum *O-*glycosylated proteins as biomarkers for detection of early breast cancer. However, the difference in fold changes between stage 0 and stage I (compared to the controls) was not statistically significant.

**Table 2:** CGB lectin isolated proteins of altered abundance.

Band^‡^	Protein Name	MS/MS Search score	Sequence Coverage (%)	Theoretical Mass (Da)	Observed Mass (Da)
b1	Proteoglycan 4	67	8	55460	317592
b2	Plasma Protease C1 Inhibitor	444	31	55734	95156
b3	Plasma Protease C1 Inhibitor	708	34	55734	85124
b4	Not identified	-	-	-	37715

^‡^Refers to excised electrophoretic bands as depicted in Fig 3.

### Western blot analysis of plasma protease C1 inhibitor

**Fig 5:**
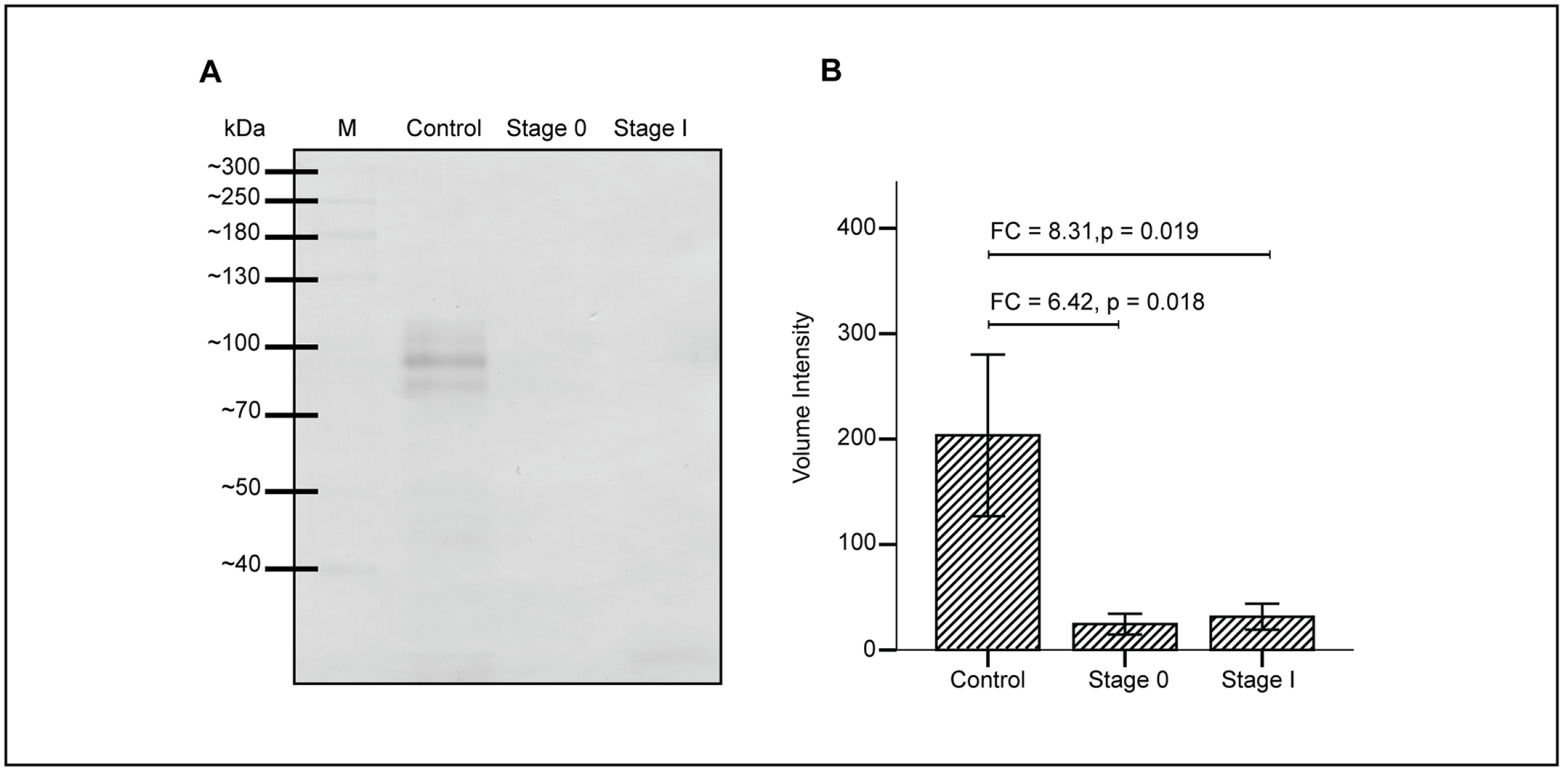
Western blot analysis of protease C1 inhibitor in serum PCA isolates of normal control women and breast cancer patients. CGB lectin bound proteins of serum PCA isolates were transferred onto nitrocellulose membrane and blotted using rabbit anti-plasma protease C1 inhibitor (a). Intensities of the bands were analysed using the GelAnalyzer software and volume intensity was compared across the three groups of samples analysed (b).

To validate the levels of protease C1 inhibitor in serum PCA isolates collected after CGB lectin precipitation, western blot analysis was performed using rabbit anti-plasma protease C1 inhibitor (Fig 5, panel (a)). Similar differential intensities of protease C1 inhibitor bands which correspond to the intensities of the peptide bands that were previously detected in SDS-PAGE (Fig 3) were observed in the blot. The levels of plasma protease C1 inhibitor from western blot analysis were significantly lowered in stage 0 and stage I breast cancer patients compared to those of the controls (Fig 5, panel (b)).

## Discussion

In the present study, we described the use of PCA to enrich mucin-type glycoproteins and further improve detection of trace amounts of the heavily *O-*glycosylated proteins in serum samples. In our previous report, we have reported the development of a sensitive assay to measure mucin-type *O-*glycosylated proteins in serum samples [10]. Since the developed assay uses CGB lectin, which is known to interact with serum IgA1 to capture *O-*glycosylated proteins, the low-abundant mucin-type glycoproteins may have lesser chance to bind to the pre-coated lectin due to presence of large amounts of IgA1 in the serum. To counter this problem, we have used a traditional method to isolate heavily glycosylated proteins using PCA. The method, which was first reported by Winzler et al. (1948) to isolate mucoproteins from plasma, precipitates non and minimally-glycosylated proteins, leaving those highly glycosylated proteins in the supernatant [11].

Among the heavily glycosylated serum proteins retained in PCA, alpha-1-acid glycoprotein is most abundant. This serum glycoprotein, whose glycans structures are clearly defined [17], makes up about 70% of the total glycoproteins enriched using the method [18]. Aside from alpha-1-acid glycoprotein, other heavily glycosylated serum proteins such as hemopexin, alpha-1-acid glycoprotein 2, Zn-alpha-2-glycoprotein and haptoglobin were also reported to be solubilized in PCA [12]. In the present proteomics study, however, Zn-alpha-2-glycoprotein and haptoglobin were not identified among the glycoproteins that were retained in PCA although 10 other glycoproteins which were not earlier reported, i.e., plasma protease C1 inhibitor, alpha-1-antitrypsin, beta-2-glycoprotein 1, prothrombin, transthyretin, protein AMBP, apolipoprotein A-IV, complement C4-A/B, corticosteroid binding globulin and coagulation factor V, were additionally detected. The data of our study generally demonstrated the high capability of PCA to enrich substantial amounts of *O-*glycosylated proteins from serum samples. PCA treatment of serum was able to enhance the detection limit of sandwich ELLA, an assay used to measure *O-*glycosylated proteins, by 14-fold.

The data generated from our asialoBSM recovery analysis further demonstrated that PCA was able to isolate over 90% of heavily glycosylated proteins in serum samples. Our analysis further demonstrated that the PCA enrichment method was able to remove IgA1 as no immunoglobulins were detected in serum PCA isolates. Hence, this has allowed our sandwich ELLA to detect other low-abundant *O-*glycosylated proteins in the serum. Clearly, these experimental findings indicate that PCA is not only capable of enriching *O-*glycosylated serum proteins but also has the ability to unmask presence of the low abundant glycoproteins that are not usually detected using other methods.

Tumour cells of cancer patients are known to actively release many different types of *O-*glycosylated proteins into the blood circulation [5, 19, 20]. In the present study, ELLA performed on serum samples of patients with stage 0 and stage I breast cancer demonstrated highly elevated levels of total *O-*glycosylated proteins compared to those obtained from controls. When similar experiments were repeated using PCA enriched fractions of serum samples of the same groups of subjects, the levels of *O-*glycosylated proteins also appeared highly elevated in both stage 0 and stage I breast cancer patients despite that IgA1 had been removed from the supernatants. This is indicative of presence of additional heavily *O-*glycosylated proteins in the serum PCA isolates that might have been captured by ELLA in absence of IgA1. Substantial improvement of sensitivity and specificity of sandwich ELLA to detect both stage 0 and stage I breast cancer was also achieved using the PCA-enriched serum samples. Hence, we have generally observed the elevated levels of total *O*-glycosylated proteins in breast cancer patients using sandwich ELLA. Our next step was to determine specific *O*-glycosylated proteins that might have contributed to the different levels observed.

Several methods, including lectin affinity chromatography [21] and lectin blotting [22, 23], have earlier been used in studies identifying the composition of *O-*glycosylated proteins in sample fractions. However, these methods require substantial glycoprotein samples. In view of the limited amounts of pooled serum sample fractions, we have adopted the CGB-lectin-conjugated beads to capture the heavily *O-*glycosylated proteins before analysing their protein compositions via mass spectrometry. In this method, all *O-*glycosylated proteins that were bound to the lectin conjugated beads were solubilized in Laemmli buffer before being resolved by SDS-PAGE.

When comparative analysis of the PCA-enriched *O-*glycosylated proteins of the controls and patients with stage 0 and stage I breast cancer was performed, different electrophoretic profiles were clearly generated. Densitometry of the bands and analysis by mass spectrometry and database search demonstrated two identified serum proteins with more than 1.5-fold difference in abundance. While, the intensity of proteoglycan 4 appeared significantly elevated in serum PCA isolates of both stage 0 and stage I breast cancer samples, the bands of plasma protease C1 inhibitor proteins appeared significantly more intense in those of the controls. Hence, proteoglycan 4 may be one of the heavily *O*-glycosylated proteins whose levels were earlier shown to be elevated in the serum samples of patients with breast cancer.

Proteoglycan 4 is a secreted mucinous glycoprotein found highly in abundance in the synovial fluid of mammalian diarthrodal joints [24]. The glycoprotein, which is also known as lubricin, functions as a boundary lubricant at the articular cartilage surface [25, 26]. However, proteoglycan 4 is not solely expressed in the articular tissues. Studies have shown that the glycoprotein is also expressed in tissues of the heart, liver, bone [27], eye [28] and breast [29] as well as in the blood circulation [30]. This suggests that it may have other functions aside from lubrication of joints. One of the unique features of this protein is that it contains multiple *O*-linked oligosaccharide chains, which comprise mostly core 1 (Galβ1,3GalNAcα1-*O*-Ser/Thr) and core 2 (Galβ1,3[GlcNAcβ1,6]GalNAcα1-*O*-Ser/Thr) structures attached to a serine/threonine/proline rich (STP-rich) domain [31]. Interestingly, a recent study capitalizing on the advances in glycoproteomics using innovative cancer cell genetic engineering [32, 33] has clearly identified proteoglycan 4 as one of a variety of *O*-glycoproteins with the truncated STn (NeuAcα2-6GalNAcα1-*O*-Ser/Thr) glycoform in the sera of gastric carcinoma patients [34]. However, several attempts made to validate its altered levels in serum samples of breast cancer patients using different antisera so far had not been successful.

Plasma protease C1 inhibitor belongs to the serine proteinase inhibitor (serpin) family. Also known as serpin G1, it is active against complement C1s and C1r, kallikrein, and coagulation factor XIIa, and hence its roles as a major inhibitor of complement and in regulating physiological pathways such as blood coagulation, fibrinolysis and the generation of kinins [35]. Lower levels of plasma protease C1 inhibitor often relate to a rare genetic disease known as hereditary angioedema [36, 37]. Previously, the plasma protein has been shown to be elevated in 64 patients with several different malignancies using radial immunodiffusion [38]. In the present study, however, the intensity of plasma protease C1 inhibitor bands generated from serum PCA isolates of normal control women appeared more intense than those generated from both stage 0 and stage I breast cancer patients. Although this generally reflects lower abundance of the protease inhibitor in the breast cancer patients, it could also be because of their differences in *O-*glycosylation and/or solubility in PCA. Plasma protease C1 inhibitor is known to be heavily glycosylated with both the *N-* and *O-*linked oligosaccharide moieties [39], and alteration of glycan structures of glycoproteins is also not an uncommon phenomenon in malignancies [19, 20, 40]. In this study, the same reduced expression of protease C1 inhibitor in patients with early stages of breast cancer relative to the controls was further observed in a western blot experiment using antisera to detect the serum glycoprotein.

In view of the reciprocal trend of altered abundance of proteoglycan 4 and plasma protease C1 inhibitor between the breast cancer patients and cancer free women, the two serum *O-*glycosylated proteins stand to offer as strong complementary biomarker candidates for detection of early breast cancer although this requires validation in clinically representative populations using simpler high-throughput methods. At these early stages of breast cancer, treatment is generally more effective. In the case of stage 0 or carcinoma in situ, curative treatments precluding chemotherapy can be instituted.

## Conclusion

In summary, we were able to demonstrate the capability of PCA to enrich sufficient amounts of *O*-glycosylated proteins from the human serum and analysed their protein composition. The use of PCA also improved detection of serum *O*-glycosylated proteins using an earlier developed sandwich ELLA method. By subjecting CGB lectin-captured glycoprotein fractions of serum PCA isolates of the stage 0 and stage I breast cancer patients and those of controls to SDS-PAGE, we were able to further show that the intensities of proteoglycan 4 and plasma protease C1 inhibitor bands were differently altered. Our data is highly suggestive of their potential roles as complementary tumour markers for screening of early breast cancer and emphasizes the need for their validation in large-scale and clinically representative populations.

## Supporting Information

S1 Appendix Raw data of Figures and Tables. (ZIP)

## Abbreviations

BSM: bovine submaxillary mucin
ELLA: enzyme-linked lectin assay
LC/MS: liquid chromatography-mass spectrometry
PCA: perchloric acid
SDS-PAGE: sodium dodecyl sulphate-polyacrylamide gel electrophoresis
