# Supplementary material for: Unmasking Heavily O-Glycosylated Serum Proteins Using Perchloric Acid: Identification of Serum Proteoglycan 4 and Protease C1 Inhibitor as Molecular Indicators for Screening of Breast Cancer
Source: PLoS One. 2016 Feb 18;11(2):e0149551. doi: 10.1371/journal.pone.0149551 (PMC4758733; doi:10.1371/journal.pone.0149551)
Supplement: S1 Appendix — (ZIP) [file pone.0149551.s001.zip › Supporting information/S5 Table 1.pdf]

# Spectrum Mill - Protein Peptide/Summary

**Results Shown Filtered by Validation Category: valid**
**Data Directory: msdataSM\test**
hit table read 75 files - SpecFeatures read
valid hits read from tagSummary file - **Files:** 75 **Hits:** 78
beginning to assemble proteins .... proteins assembled 0.002045 sec
proteins filtered by distinct peptides 0.000617 sec
proteins filtered by score
calculated protein coverage maps 0.013663 sec
beginning to roll up proteins into groups ... . proteins rolled up into groups 0.001736 sec
protein groups ready for display
proteinGroupingMethod: oneSharedPeptideSubgrouped 14 Proteins listed

| Group (#) | Subgroup (#) | Spectra (#) | Distinct Peptides (#) | Distinct Summed MS/MS Search Score | % AA Coverage | Total Protein Spectral Intensity | Protein MW (Da) | Species | Database Accession # | Protein Name |
|---|---|---|---|---|---|---|---|---|---|---|
| 1 | 1.1 | 12 | 10 | 194.37 | 26.6 | 7.19e+006 | 55381.4 | HUMAN | P05155P05155 | Plasma protease C1 inhibitor |
| 2 | 2.1 | 9 | 9 | 174.65 | 31.1 | 7.85e+006 | 46906.8 | HUMAN | P01009P01009 | Alpha-1-antitrypsin |
| 3 | 3.1 | 14 | 5 | 104.45 | 27.8 | 7.54e+006 | 23739.3 | HUMAN | P02763P02763 | Alpha-1-acid glycoprotein 1 |
| 4 | 4.1 | 5 | 5 | 96.15 | 27.5 | 2.39e+006 | 39609.7 | HUMAN | P02749P02749 | Beta-2-glycoprotein 1 |
| 5 | 5.1 | 5 | 4 | 81.88 | 10.4 | 1.91e+006 | 71519.0 | HUMAN | P00734P00734 | Prothrombin |
| 6 | 6.1 | 4 | 4 | 79.74 | 12.7 | 5.23e+006 | 52417.1 | HUMAN | P02790P02790 | Hemopexin |
| 7 | 7.1 | 4 | 4 | 76.93 | 48.2 | 8.98e+005 | 16000.8 | HUMAN | P02766P02766 | Transthyretin |
| 8 | 8.1 | 4 | 3 | 55.81 | 11.3 | 2.56e+006 | 39911.7 | HUMAN | P02760P02760 | Protein AMBP |
| 9 | 9.1 | 2 | 2 | 45.18 | 7.3 | 1.87e+005 | 45398.2 | HUMAN | P06727P06727 | Apolipoprotein A-IV |
| 10 | 10.1 | 12 | 2 | 40.12 | 14.4 | 3.79e+006 | 23887.5 | HUMAN | P19652P19652 | Alpha-1-acid glycoprotein 2 |
| 11 | 11.1 | 2 | 2 | 34.82 | 0.8 | 0.00e+000 | 194379.5 | HUMAN | P0C0L4P0C0L4 | Complement C4-A |
| 11 | 11.1 | 2 | 2 | 34.82 | 0.8 | 0.00e+000 | 194288.4 | HUMAN | P0C0L5P0C0L5 | Complement C4-B |
| 12 | 12.1 | 1 | 1 | 19.52 | 3.7 | 2.58e+005 | 45311.2 | HUMAN | P08185P08185 | Corticosteroid-binding globulin |
| 13 | 13.1 | 1 | 1 | 18.51 | 0.5 | 2.42e+005 | 252839.9 | HUMAN | P12259P12259 | Coagulation factor V |
| 14 | 14.1 | 1 | 1 | 17.09 | 7 | 6.14e+005 | 14179.2 | HUMAN | P02775P02775 | Platelet basic protein |
| **Totals:** | 78 | 55 | | | | | | | | |

| Group (#) | Subgroup (#) | Spectra (#) | Distinct Peptides (#) | Distinct Summed MS/MS Search Score | % AA Coverage | Total Protein Spectral Intensity | Protein MW (Da) | Species | Database Accession # | Protein Name |
|---|---|---|---|---|---|---|---|---|---|---|
| 1 | 1.1 | 12 | 10 | 194.37 | 26.6 | 7.19e+006 | 55381.4 | HUMAN | P05155P05155 | Plasma protease C1 inhibitor ▼ |

| # | Filename | z | Score | Local FDR (%) | Global FDR (%) | FDR Search # | Fwd-Rev Score | SPI (%) | Spectrum Intensity | Sequence | Peptide pI |
|---|---|---|---|---|---|---|---|---|---|---|---|
| 1 | PCA_Supt_Prot.2312.2312.2 | 2 | 18.13 | <0.1% | <0.1% | 1 | 1.02 | 83.083.0 | 1.64e+004 | (K)DFTCVHQALK(G) | 6.74 |
| 2 | PCA_Supt_Prot.2324.2328.2 | 2 | 17.58 | 8% | 1% | 1 | 5.86 | 85.985.9 | 3.10e+005 | (K)DFTCVHQALK(G) | 6.74 |
| 3 | PCA_Supt_Prot.2353.2358.2 | 2 | 17.78 | 8% | 2% | 1 | 4.45 | 88.988.9 | 4.10e+004 | (K)LYHAFSAMK(K) | 8.60 |
| 4 | PCA_Supt_Prot.2464.2468.2 | 2 | 17.48 | 8% | 1% | 1 | 1.37 | 93.593.5 | 6.65e+005 | (R)LLDSLPSDTR(L) | 4.21 |
| 5 | PCA_Supt_Prot.3236.3243.2 | 2 | 17.77 | 8% | 2% | 1 | 5.23 | 84.684.6 | 1.51e+005 | (K)TNLESILSYPK(D) | 5.66 |
| 6 | PCA_Supt_Prot.3318.3326.2 | 2 | 21.43 | <0.1% | <0.1% | 1 | 10.92 | 92.992.9 | 2.90e+005 | (R)LEDMEQALSPSVFK(A) | 4.14 |
| 7 | PCA_Supt_Prot.3403.3411.2 | 2 | 19.86 | <0.1% | <0.1% | 1 | 8.05 | 95.395.3 | 7.72e+005 | (K)GVTSVSQIFHSPDLAIR(D) | 6.74 |
| 8 | PCA_Supt_Prot.3417.3429.2 | 2 | 19.19 | <0.1% | <0.1% | 1 | 8.97 | 80.080.0 | 3.07e+005 | (K)GVTSVSQIFHSPDLAIR(D) | 6.74 |
| 9 | PCA_Supt_Prot.3685.3804.2 | 2 | 21.47 | <0.1% | <0.1% | 1 | 8.09 | 93.993.9 | 1.63e+006 | (R)LVLLNAIYLSAK(W) | 8.59 |
| 10 | PCA_Supt_Prot.4304.4309.3 | 3 | 19.40 | <0.1% | <0.1% | 1 | 5.55 | 92.192.1 | 1.49e+006 | (R)LLDSLPSDTRLVLLNAIYLSAK(W) | 5.96 |
| 11 | PCA_Supt_Prot.4632.4639.3 | 3 | 21.19 | <0.1% | <0.1% | 1 | 9.90 | 94.894.8 | 7.70e+005 | (R)TLLVFEVQQPFLFVLWDQQHK(F) | 5.29 |
| 12 | PCA_Supt_Prot.5587.5591.3 | 3 | 19.86 | <0.1% | <0.1% | 1 | 7.52 | 90.990.9 | 7.51e+005 | (K)KVETNMAFSPFSIASLLTQVLLGAGENTK(T) | 6.14 |

| Group (#) | Subgroup (#) | Spectra (#) | Distinct Peptides (#) | Distinct Summed MS/MS Search Score | % AA Coverage | Total Protein Spectral Intensity | Protein MW (Da) | Species | Database Accession # | Protein Name |
|---|---|---|---|---|---|---|---|---|---|---|
| 2 | 2.1 | 9 | 9 | 174.65 | 31.1 | 7.85e+006 | 46906.8 | HUMAN | P01009P01009 | Alpha-1-antitrypsin ▼ |

| # | Filename | z | Score | Local FDR (%) | Global FDR (%) | FDR Search # | Fwd-Rev Score | SPI (%) | Spectrum Intensity | Sequence | Peptide pI |
|---|---|---|---|---|---|---|---|---|---|---|---|
| 1 | PCA_Supt_Prot.2735.2740.2 | 2 | 18.78 | <0.1% | <0.1% | 1 | 4.51 | 92.792.7 | 1.32e+006 | (K)LSITGTYDLK(S) | 5.83 |
| 2 | PCA_Supt_Prot.3007.3012.3 | 3 | 17.77 | 8% | 2% | 1 | 4.71 | 87.787.7 | 2.70e+005 | (K)KLYHSEAFTVNFGDTEEAK(K) | 4.83 |
| 3 | PCA_Supt_Prot.3152.3158.3 | 3 | 16.95 | 66% | 7% | 1 | 4.57 | 90.390.3 | 9.19e+005 | (K)VFSNGADLSGVTEEAPLKLSK(A) | 4.68 |
| 4 | PCA_Supt_Prot.3508.3521.2 | 2 | 20.73 | <0.1% | <0.1% | 1 | 6.54 | 92.092.0 | 3.22e+005 | (K)LSSWVLLMK(Y) | 8.75 |
| 5 | PCA_Supt_Prot.3637.3644.3 | 3 | 20.00 | <0.1% | <0.1% | 1 | 4.84 | 92.592.5 | 7.11e+005 | (R)TLNQPDSQLQLTTGNGLFLSEGLK(L) | 4.37 |

| | | | | | | | | | | | |
|---|---|---|---|---|---|---|---|---|---|---|---|
| 6 | PCA_Supt_Prot.4091.4095.3 | 3 | 18.96 | <0.1% | <0.1% | 1 | 6.40 | 89.489.4 | 8.40e+004 | (K)LSITGTYDLKSVLGQLGITK(V) | 8.50 |
| 7 | PCA_Supt_Prot.4148.4154.2 | 2 | 22.68 | <0.1% | <0.1% | 1 | 9.18 | 93.793.7 | 5.49e+005 | (K)ITPNLAEFAFSLYR(Q) | 6.00 |
| 8 | PCA_Supt_Prot.4740.4850.2 | 2 | 20.98 | <0.1% | <0.1% | 1 | 10.74 | 94.394.3 | 2.94e+006 | (R)DTVFALVNYIFFK(G) | 5.83 |
| 9 | PCA_Supt_Prot.5245.5248.3 | 3 | 17.80 | 8% | 2% | 1 | 3.12 | 89.989.9 | 7.28e+005 | (K)IVDLVKELDRDTVFALVNYIFFK(G) | 4.68 |

| Group (#) | Subgroup (#) | Spectra (#) | Distinct Peptides (#) | Distinct Summed MS/MS Search Score | % AA Coverage | Total Protein Spectral Intensity | Protein MW (Da) | Species | Database Accession # | Protein Name |
|---|---|---|---|---|---|---|---|---|---|---|
| 3 | 3.1 | 14 | 5 | 104.45 | 27.8 | 7.54e+006 | 23739.3 | HUMAN | P02763P02763 | Alpha-1-acid glycoprotein 1 ▼ |

| # | Filename | z | Score | Local FDR (%) | Global FDR (%) | FDR Search # | Fwd-Rev Score | SPI (%) | Spectrum Intensity | Sequence | Peptide pI |
|---|---|---|---|---|---|---|---|---|---|---|---|
| 1 | PCA_Supt_Prot.2730.2777.2 | 2 | 21.42 | <0.1% | <0.1% | 1 | 9.37 | 92.192.1 | 8.00e+005 | (K)NWGLSVYADKPETTK(E) | 6.07 |
| 2 | PCA_Supt_Prot.2894.2900.2 | 2 | 19.18 | <0.1% | <0.1% | 1 | 9.48 | 81.881.8 | 7.46e+005 | (K)TYMLAFDVNDEK(N) | 4.03 |
| 3 | PCA_Supt_Prot.3110.3312.3 | 3 | 17.78 | 8% | 2% | 1 | 17.78 | 80.580.5 | 2.61e+003 | (R)YVGGQEHFAHLLILR(D) | 6.92 |
| 4 | PCA_Supt_Prot.3111.3118.2 | 2 | 27.59 | <0.1% | <0.1% | 1 | 14.66 | 98.198.1 | 2.17e+006 | (R)YVGGQEHFAHLLILR(D) | 6.92 |
| 5 | PCA_Supt_Prot.3230.3230.2 | 2 | 21.24 | <0.1% | <0.1% | 1 | 8.96 | 85.185.1 | 1.50e+005 | (R)YVGGQEHFAHLLILR(D) | 6.92 |
| 6 | PCA_Supt_Prot.3624.3632.3 | 3 | 17.31 | 66% | 4% | 1 | 5.22 | 86.086.0 | 3.39e+005 | (K)TYMLAFDVNDEKNWGLSVYADKPETTK(E) | 4.44 |
| 7 | PCA_Supt_Prot.3665.3684.2 | 2 | 17.61 | 8% | 1% | 1 | 9.13 | 83.883.8 | 3.67e+005 | (K)EQLGEFYEALDCLR(I) | 4.00 |
| 8 | PCA_Supt_Prot.3740.3747.2 | 2 | 17.03 | 66% | 7% | 1 | 6.92 | 79.479.4 | 3.32e+005 | (K)EQLGEFYEALDCLR(I) | 4.00 |
| 9 | PCA_Supt_Prot.3761.3867.2 | 2 | 17.52 | 8% | 1% | 1 | 9.70 | 83.883.8 | 2.28e+004 | (K)EQLGEFYEALDCLR(I) | 4.00 |
| 10 | PCA_Supt_Prot.3778.3783.2 | 2 | 18.31 | <0.1% | <0.1% | 1 | 5.64 | 88.588.5 | 3.96e+005 | (K)EQLGEFYEALDCLR(I) | 4.00 |
| 11 | PCA_Supt_Prot.3931.3940.2 | 2 | 18.82 | <0.1% | <0.1% | 1 | 9.46 | 81.381.3 | 7.98e+004 | (K)EQLGEFYEALDCLR(I) | 4.00 |
| 12 | PCA_Supt_Prot.3986.3990.2 | 2 | 18.27 | <0.1% | <0.1% | 1 | 8.02 | 82.582.5 | 1.55e+006 | (K)EQLGEFYEALDCLR(I) | 4.00 |
| 13 | PCA_Supt_Prot.4041.4049.2 | 2 | 18.88 | <0.1% | <0.1% | 1 | 9.41 | 84.384.3 | 3.63e+005 | (K)EQLGEFYEALDCLR(I) | 4.00 |
| 14 | PCA_Supt_Prot.4217.4222.2 | 2 | 18.95 | <0.1% | <0.1% | 1 | 8.97 | 97.097.0 | 2.23e+005 | (K)EQLGEFYEALDCLR(I) | 4.00 |

| Group (#) | Subgroup (#) | Spectra (#) | Distinct Peptides (#) | Distinct Summed MS/MS Search Score | % AA Coverage | Total Protein Spectral Intensity | Protein MW (Da) | Species | Database Accession # | Protein Name |
|---|---|---|---|---|---|---|---|---|---|---|
| 4 | 4.1 | 5 | 5 | 96.15 | 27.5 | 2.39e+006 | 39609.7 | HUMAN | P02749P02749 | Beta-2-glycoprotein 1 ▼ |

| # | Filename | z | Score | Local FDR (%) | Global FDR (%) | FDR Search # | Fwd-Rev Score | SPI (%) | Spectrum Intensity | Sequence | Peptide pI |
|---|---|---|---|---|---|---|---|---|---|---|---|
| 1 | PCA_Supt_Prot.2803.2808.3 | 3 | 17.56 | 8% | 1% | 1 | 9.07 | 85.385.3 | 4.52e+005 | (K)ATFGCHDGYSLDGPEEIECTK(L) | 4.17 |
| 2 | PCA_Supt_Prot.2865.2880.3 | 3 | 18.68 | <0.1% | <0.1% | 1 | 8.21 | 95.195.1 | 3.62e+005 | (K)TFYEPGEEITYSCKPGYVSR(G) | 4.78 |
| 3 | PCA_Supt_Prot.3391.3397.2 | 2 | 20.31 | <0.1% | <0.1% | 1 | 8.29 | 86.686.6 | 2.82e+005 | (R)VCPFAGILENGAVR(Y) | 5.97 |
| 4 | PCA_Supt_Prot.4260.4264.2 | 2 | 19.80 | <0.1% | <0.1% | 1 | 6.56 | 89.889.8 | 6.77e+005 | (K)FICPLTGLWPINTLK(C) | 8.75 |
| 5 | PCA_Supt_Prot.4286.4292.3 | 3 | 19.80 | <0.1% | <0.1% | 1 | 4.40 | 91.191.1 | 6.21e+005 | (K)WSPELPVCAPIICPPPSIPTFATLR(V) | 6.00 |

| Group (#) | Subgroup (#) | Spectra (#) | Distinct Peptides (#) | Distinct Summed MS/MS Search Score | % AA Coverage | Total Protein Spectral Intensity | Protein MW (Da) | Species | Database Accession # | Protein Name |
|---|---|---|---|---|---|---|---|---|---|---|
| 5 | 5.1 | 5 | 4 | 81.88 | 10.4 | 1.91e+006 | 71519.0 | HUMAN | P00734P00734 | Prothrombin ▼ |

| # | Filename | z | Score | Local FDR (%) | Global FDR (%) | FDR Search # | Fwd-Rev Score | SPI (%) | Spectrum Intensity | Sequence | Peptide pI |
|---|---|---|---|---|---|---|---|---|---|---|---|
| 1 | PCA_Supt_Prot.2703.2713.3 | 3 | 20.50 | <0.1% | <0.1% | 1 | 7.06 | 93.293.2 | 1.99e+005 | (K)LAACLEGNCAEGLGTNYR(G) | 4.53 |
| 2 | PCA_Supt_Prot.2708.2715.2 | 2 | 24.07 | <0.1% | <0.1% | 1 | 12.89 | 92.692.6 | 1.51e+005 | (K)LAACLEGNCAEGLGTNYR(G) | 4.53 |
| 3 | PCA_Supt_Prot.2728.2728.2 | 2 | 17.85 | 8% | 2% | 1 | 3.65 | 81.381.3 | 7.04e+005 | (R)SGIECQLWR(S) | 5.72 |
| 4 | PCA_Supt_Prot.2907.2913.3 | 3 | 19.41 | <0.1% | <0.1% | 1 | 7.52 | 88.488.4 | 2.00e+005 | (R)SEGSSVNLSPPLEQCVPDR(G) | 4.14 |
| 5 | PCA_Supt_Prot.3341.3341.3 | 3 | 20.55 | <0.1% | <0.1% | 1 | 7.70 | 95.795.7 | 6.53e+005 | (R)LAVTTHGLPCLAWASAQAK(A) | 8.76 |

| Group (#) | Subgroup (#) | Spectra (#) | Distinct Peptides (#) | Distinct Summed MS/MS Search Score | % AA Coverage | Total Protein Spectral Intensity | Protein MW (Da) | Species | Database Accession # | Protein Name |
|---|---|---|---|---|---|---|---|---|---|---|
| 6 | 6.1 | 4 | 4 | 79.74 | 12.7 | 5.23e+006 | 52417.1 | HUMAN | P02790P02790 | Hemopexin ▼ |

| # | Filename | z | Score | Local FDR (%) | Global FDR (%) | FDR Search # | Fwd-Rev Score | SPI (%) | Spectrum Intensity | Sequence | Peptide pI |
|---|---|---|---|---|---|---|---|---|---|---|---|
| 1 | PCA_Supt_Prot.3047.3054.2 | 2 | 19.68 | <0.1% | <0.1% | 1 | 5.66 | 85.085.0 | 1.45e+006 | (K)NFPSPVDAAFR(Q) | 5.84 |
| 2 | PCA_Supt_Prot.3069.3074.2 | 2 | 18.73 | <0.1% | <0.1% | 1 | 5.83 | 89.989.9 | 5.52e+005 | (R)YYCFQGNQFLR(F) | 8.59 |
| 3 | PCA_Supt_Prot.3249.3256.3 | 3 | 17.07 | 66% | 8% | 1 | 5.59 | 85.885.8 | 1.50e+006 | (K)SGAQATWTELPWPHEK(V) | 5.38 |
| 4 | PCA_Supt_Prot.3650.3658.3 | 3 | 24.26 | <0.1% | <0.1% | 1 | 10.18 | 97.197.1 | 1.72e+006 | (K)LLQDEFPGIPSPLDAAVECHR(G) | 4.31 |

| Group | Subgroup | Spectra | Distinct | Distinct | % AA | Total Protein | Database |
|---|---|---|---|---|---|---|---|

| (#) | (#) | (#) | Peptides (#) | Summed MS/MS Search Score | Coverage | Spectral Intensity | Protein MW (Da) | Species | Accession # | Protein Name |
|---|---|---|---|---|---|---|---|---|---|---|
| 7 | 7.1 | 4 | 4 | 76.93 | 48.2 | 8.98e+005 | 16000.8 | HUMAN | P02766P02766 | Transthyretin ▼ |

| # | Filename | z | Score | Local FDR (%) | Global FDR (%) | FDR Search # | Fwd-Rev Score | SPI (%) | Spectrum Intensity | Sequence | Peptide pI |
|---|---|---|---|---|---|---|---|---|---|---|---|
| 1 | PCA_Supt_Prot.2775.2781.2 | 2 | 21.76 | <0.1% | <0.1% | 1 | 13.68 | 92.992.9 | 2.88e+005 | (K)AADDTWEPFASGK(T) | 4.03 |
| 2 | PCA_Supt_Prot.3068.3076.3 | 3 | 17.61 | 8% | 1% | 1 | 9.06 | 86.286.2 | 4.19e+005 | (R)GSPAINVAVHVFR(K) | 9.75 |
| 3 | PCA_Supt_Prot.3097.3097.3 | 3 | 19.67 | <0.1% | <0.1% | 1 | 5.46 | 100.0100.0 | 1.39e+005 | (K)ALGISPFHEHAEVVFTANDSGPR(R) | 5.27 |
| 4 | PCA_Supt_Prot.3919.3925.3 | 3 | 17.89 | 8% | 2% | 1 | 4.63 | 87.387.3 | 5.14e+004 | (R)YTIAALLSPYSYSTTAVVTNPK(E) | 8.43 |

| Group (#) | Subgroup (#) | Spectra (#) | Distinct Peptides (#) | Distinct Summed MS/MS Search Score | % AA Coverage | Total Protein Spectral Intensity | Protein MW (Da) | Species | Database Accession # | Protein Name |
|---|---|---|---|---|---|---|---|---|---|---|
| 8 | 8.1 | 4 | 3 | 55.81 | 11.3 | 2.56e+006 | 39911.7 | HUMAN | P02760P02760 | Protein AMBP ▼ |

| # | Filename | z | Score | Local FDR (%) | Global FDR (%) | FDR Search # | Fwd-Rev Score | SPI (%) | Spectrum Intensity | Sequence | Peptide pI |
|---|---|---|---|---|---|---|---|---|---|---|---|
| 1 | PCA_Supt_Prot.2667.2671.2 | 2 | 17.77 | 8% | 2% | 1 | 5.31 | 89.989.9 | 5.00e+005 | (R)TVAACNLPIVR(G) | 9.41 |
| 2 | PCA_Supt_Prot.3085.3085.2 | 2 | 19.08 | <0.1% | <0.1% | 1 | 8.28 | 76.976.9 | 1.99e+005 | (R)GECVPGEQEPEPILIPR(V) | 4.09 |
| 3 | PCA_Supt_Prot.4000.4003.2 | 2 | 18.96 | <0.1% | <0.1% | 1 | 4.64 | 81.281.2 | 4.28e+005 | (R)AFIQLWAFDAVK(G) | 5.88 |
| 4 | PCA_Supt_Prot.4027.4033.2 | 2 | 18.37 | <0.1% | <0.1% | 1 | 7.27 | 82.882.8 | 1.44e+006 | (R)AFIQLWAFDAVK(G) | 5.88 |

| Group (#) | Subgroup (#) | Spectra (#) | Distinct Peptides (#) | Distinct Summed MS/MS Search Score | % AA Coverage | Total Protein Spectral Intensity | Protein MW (Da) | Species | Database Accession # | Protein Name |
|---|---|---|---|---|---|---|---|---|---|---|
| 9 | 9.1 | 2 | 2 | 45.18 | 7.3 | 1.87e+005 | 45398.2 | HUMAN | P06727P06727 | Apolipoprotein A-IV ▼ |

| # | Filename | z | Score | Local FDR (%) | Global FDR (%) | FDR Search # | Fwd-Rev Score | SPI (%) | Spectrum Intensity | Sequence | Peptide pI |
|---|---|---|---|---|---|---|---|---|---|---|---|
| 1 | PCA_Supt_Prot.2111.2114.2 | 2 | 21.03 | <0.1% | <0.1% | 1 | 7.57 | 90.190.1 | 1.43e+004 | (R)SLAPYAQDTQEK(L) | 4.37 |
| 2 | PCA_Supt_Prot.3257.3263.3 | 3 | 24.15 | <0.1% | <0.1% | 1 | 9.62 | 97.797.7 | 1.73e+005 | (K)SLAELGGHLDQQVEEFR(R) | 4.40 |

| Group (#) | Subgroup (#) | Spectra (#) | Distinct Peptides (#) | Distinct Summed MS/MS Search Score | % AA Coverage | Total Protein Spectral Intensity | Protein MW (Da) | Species | Database Accession # | Protein Name |
|---|---|---|---|---|---|---|---|---|---|---|
| 10 | 10.1 | 12 | 2 | 40.12 | 14.4 | 3.79e+006 | 23887.5 | HUMAN | P19652P19652 | Alpha-1-acid glycoprotein 2 ▼ |

| # | Filename | z | Score | Local FDR (%) | Global FDR (%) | FDR Search # | Fwd-Rev Score | SPI (%) | Spectrum Intensity | Sequence | Peptide pI |
|---|---|---|---|---|---|---|---|---|---|---|---|
| 1 | PCA_Supt_Prot.3211.3218.2 | 2 | 18.97 | <0.1% | <0.1% | 1 | 7.59 | 89.489.4 | 3.97e+005 | (K)TLMFGSYLDDEK(N) | 4.03 |
| 2 | PCA_Supt_Prot.3955.4065.3 | 3 | 18.58 | <0.1% | <0.1% | 1 | 4.84 | 91.591.5 | 7.32e+004 | (K)EQLGEFYEALDCLCIPR(S) | 4.00 |
| 3 | PCA_Supt_Prot.3959.4062.3 | 3 | 17.27 | 66% | 5% | 1 | 5.38 | 94.494.4 | 1.52e+004 | (K)EQLGEFYEALDCLCIPR(S) | 4.00 |
| 4 | PCA_Supt_Prot.3960.3964.2 | 2 | 20.40 | <0.1% | <0.1% | 1 | 12.02 | 91.891.8 | 4.50e+004 | (K)EQLGEFYEALDCLCIPR(S) | 4.00 |
| 5 | PCA_Supt_Prot.3971.3977.2 | 2 | 20.05 | <0.1% | <0.1% | 1 | 11.82 | 90.890.8 | 5.24e+005 | (K)EQLGEFYEALDCLCIPR(S) | 4.00 |
| 6 | PCA_Supt_Prot.3980.4240.3 | 3 | 17.80 | 8% | 2% | 1 | 7.64 | 84.584.5 | 5.30e+005 | (K)EQLGEFYEALDCLCIPR(S) | 4.00 |
| 7 | PCA_Supt_Prot.4004.4009.3 | 3 | 19.64 | <0.1% | <0.1% | 1 | 5.98 | 89.089.0 | 4.06e+005 | (K)EQLGEFYEALDCLCIPR(S) | 4.00 |
| 8 | PCA_Supt_Prot.4006.4010.2 | 2 | 21.15 | <0.1% | <0.1% | 1 | 10.38 | 74.374.3 | 1.32e+005 | (K)EQLGEFYEALDCLCIPR(S) | 4.00 |
| 9 | PCA_Supt_Prot.4012.4054.2 | 2 | 19.34 | <0.1% | <0.1% | 1 | 8.38 | 81.481.4 | 4.73e+005 | (K)EQLGEFYEALDCLCIPR(S) | 4.00 |
| 10 | PCA_Supt_Prot.4052.4052.3 | 3 | 17.69 | 8% | 2% | 1 | 2.88 | 82.782.7 | 8.61e+005 | (K)EQLGEFYEALDCLCIPR(S) | 4.00 |
| 11 | PCA_Supt_Prot.4205.4205.2 | 2 | 17.08 | 66% | 8% | 1 | 7.95 | 72.572.5 | 5.10e+004 | (K)EQLGEFYEALDCLCIPR(S) | 4.00 |
| 12 | PCA_Supt_Prot.4223.4228.2 | 2 | 20.08 | <0.1% | <0.1% | 1 | 10.71 | 89.989.9 | 2.81e+005 | (K)EQLGEFYEALDCLCIPR(S) | 4.00 |

| Group (#) | Subgroup (#) | Spectra (#) | Distinct Peptides (#) | Distinct Summed MS/MS Search Score | % AA Coverage | Total Protein Spectral Intensity | Protein MW (Da) | Species | Database Accession # | Protein Name |
|---|---|---|---|---|---|---|---|---|---|---|
| 11 | 11.1 | 2 | 2 | 34.82 | 0.8 | 0.00e+000 | 194379.5 | HUMAN | P0C0L4P0C0L4 | Complement C4-A ▼ |

| # | Filename | z | Score | Local FDR (%) | Global FDR (%) | FDR Search # | Fwd-Rev Score | SPI (%) | Spectrum Intensity | Sequence | Peptide pI |
|---|---|---|---|---|---|---|---|---|---|---|---|
| 1 | PCA_Supt_Prot.3315.3319.2 | 2 | 17.41 | 45% | 1% | 1 | 8.24 | 84.084.0 | 1.91e+005 | (R)EPFLSCCQFAESLR(K) | 4.53 |

| Group (#) | Subgroup (#) | Spectra (#) | Distinct Peptides (#) | Distinct Summed MS/MS Search Score | % AA Coverage | Total Protein Spectral Intensity | Protein MW (Da) | Species | Database Accession # | Protein Name |
|---|---|---|---|---|---|---|---|---|---|---|
| 12 | 12.1 | 1 | 1 | 19.52 | 3.7 | 2.58e+005 | 45311.2 | HUMAN | P08185P08185 | Corticosteroid-binding globulin ▼ |

| # | Filename | z | Score | Local FDR (%) | Global FDR (%) | FDR Search # | Fwd-Rev Score | SPI (%) | Spectrum Intensity | Sequence | Peptide pI |
|---|---|---|---|---|---|---|---|---|---|---|---|
| 1 | PCA_Supt_Prot.3639.3639.2 | 2 | 19.52 | <0.1% | <0.1% | 1 | 6.59 | 86.786.7 | 2.58e+005 | (R)GLASANVDFAFSLYK(H) | 5.83 |

| Group (#) | Subgroup (#) | Spectra (#) | Distinct Peptides (#) | Distinct Summed MS/MS Search Score | % AA Coverage | Total Protein Spectral Intensity | Protein MW (Da) | Species | Database Accession # | Protein Name |
|---|---|---|---|---|---|---|---|---|---|---|
| 13 | 13.1 | 1 | 1 | 18.51 | 0.5 | 2.42e+005 | 252839.9 | HUMAN | P12259P12259 | Coagulation factor V ▼ |

| # | Filename | z | Score | Local FDR (%) | Global FDR (%) | FDR Search # | Fwd-Rev Score | SPI (%) | Spectrum Intensity | Sequence | Peptide pI |
|---|---|---|---|---|---|---|---|---|---|---|---|
| 1 | PCA_Supt_Prot.3952.3957.2 | 2 | 18.51 | <0.1% | <0.1% | 1 | 10.50 | 89.889.8 | 2.42e+005 | (K)EFNPLVIVGLSK(D) | 6.10 |

| Group (#) | Subgroup (#) | Spectra (#) | Distinct Peptides (#) | Distinct Summed MS/MS Search Score | % AA Coverage | Total Protein Spectral Intensity | Protein MW (Da) | Species | Database Accession # | Protein Name |
|---|---|---|---|---|---|---|---|---|---|---|
| 14 | 14.1 | 1 | 1 | 17.09 | 7 | 6.14e+005 | 14179.2 | HUMAN | P02775P02775 | Platelet basic protein ▼ |

| # | Filename | z | Score | Local FDR (%) | Global FDR (%) | FDR Search # | Fwd-Rev Score | SPI (%) | Spectrum Intensity | Sequence | Peptide pI |
|---|---|---|---|---|---|---|---|---|---|---|---|
| 1 | PCA_Supt_Prot.2381.2387.2 | 2 | 17.09 | 66% | 8% | 1 | 2.72 | 86.086.0 | 6.14e+005 | (K)ICLDPDAPR(I) | 4.21 |
